# Glucocorticoid receptor gene inactivation in dopamine-innervated areas selectively decreases behavioral responses to amphetamine

**DOI:** 10.3389/fnbeh.2014.00035

**Published:** 2014-02-12

**Authors:** Sébastien Parnaudeau, Marie-louise Dongelmans, Marc Turiault, Frédéric Ambroggi, Anne-Sophie Delbes, Céline Cansell, Serge Luquet, Pier-Vincenzo Piazza, François Tronche, Jacques Barik

**Affiliations:** ^1^UMR 7224 CNRS, Physiopathologie des Maladies du Système Nerveux Central, “Gene Regulation and Adaptive Behaviors” GroupParis, France; ^2^INSERM, UMRs 952, Physiopathologie des Maladies du Système Nerveux CentralParis, France; ^3^Université Pierre et Marie Curie, Physiopathologie des Maladies du Système Nerveux CentralParis, France; ^4^Department of Psychiatry, Columbia UniversityNew York, NY, USA; ^5^Pathophysiology of Addiction, Institut National de la Santé et de la Recherche Médicale, U862, NeuroCentre MagendieBordeaux Cedex, France; ^6^Department of Neurology, Center for Integrative Neuroscience and the Ernest Gallo Clinic and Research Center, University of California at San FranciscoSan Francisco, CA, USA; ^7^Unité de Biologie Fonctionnelle et Adaptative, Sorbonne Paris Cité, UMR 8251 CNRS, Université Paris DiderotParis, France; ^8^Institut de Pharmacologie Moléculaire et Cellulaire, UMR 7275Valbonne, France

**Keywords:** glucocorticoid receptor, dopamine pathway, glutamate, amphetamine, food reward, motivation

## Abstract

The meso-cortico-limbic system, via dopamine release, encodes the rewarding and reinforcing properties of natural rewards. It is also activated in response to abused substances and is believed to support drug-related behaviors. Dysfunctions of this system lead to several psychiatric conditions including feeding disorders and drug addiction. These disorders are also largely influenced by environmental factors and in particular stress exposure. Stressors activate the corticotrope axis ultimately leading to glucocorticoid hormone (GCs) release. GCs bind the glucocorticoid receptor (GR) a transcription factor ubiquitously expressed including within the meso-cortico-limbic tract. While GR within dopamine-innervated areas drives cocaine's behavioral responses, its implication in responses to other psychostimulants such as amphetamine has never been clearly established. Moreover, while extensive work has been made to uncover the role of this receptor in addicted behaviors, its contribution to the rewarding and reinforcing properties of food has yet to be investigated. Using mouse models carrying *GR* gene inactivation in either dopamine neurons or in dopamine-innervated areas, we found that GR in dopamine responsive neurons is essential to properly build amphetamine-induced conditioned place preference and locomotor sensitization. c-Fos quantification in the nucleus accumbens further confirmed defective neuronal activation following amphetamine injection. These diminished neuronal and behavioral responses to amphetamine may involve alterations in glutamate transmission as suggested by the decreased MK801-elicited hyperlocomotion and by the hyporeactivity to glutamate of a subpopulation of medium spiny neurons. In contrast, GR inactivation did not affect rewarding and reinforcing properties of food suggesting that responding for natural reward under basal conditions is preserved in these mice.

## Introduction

Reward processing involves the meso-cortico-limbic system, which includes dopamine midbrain neurons and their projections to the caudate putamen (CPu), the nucleus accumbens (NAc), and the prefrontal cortex (PFC). Both addictive drugs and natural rewards act on these brain circuits that are likely to have evolved to motivate vital behaviors, including eating (Kelley and Berridge, 2002). Indeed, most drugs of abuse food rewards elicit an increase in DA release within the NAc (Di Chiara and Imperato, 1988; Hernandez and Hoebel, 1988) thought to participate to the encoding of rewarding and reinforcing properties of food rewards and addictive substances.

Vulnerability to abused drugs varies from one person to another. This interindividual variability most probably relies on both genetic and environmental factors, including stress exposure (Sinha, 2001). Similarly, stress exposure has also been shown to affect food intake and has been associated with feeding disorders (Torres and Nowson, 2007). Stress response triggers a large set of physiological reactions, including the activation of the hypothalamo-pituitary-adrenal (HPA) axis, ultimately leading to the secretion of glucocorticoids (GCs) by the adrenal gland in the blood flow. GCs activate two related nuclear receptors, the glucocorticoid receptor (GR) ubiquitously expressed, including within neurons of the reward circuitry, and the mineralocorticoid receptor (MR) restricted to more discrete brain regions. Both act as transcription factors, in the nucleus, to control gene expression and, at the membrane, participate to the rapid modulation of neuronal excitability and intracellular signaling cascades. During stress response MR is involved in the appraisal of novel situations whereas GR facilitates the consolidation of stress-related information (Groeneweg et al., 2011).

Clinical studies, supported by compelling animal data, underlie the central role of GCs in modulating responses to abused drugs and feeding behaviors (Marinelli and Piazza, 2002; Dallman et al., 2004; Sinha et al., 2006; Adam and Epel, 2007). For example, surgical suppression of circulating GCs in rats decreases locomotor responses to psychostimulants, an effect rescued by hormone replacement (Marinelli et al., 1997). Similarly, adrenalectomy have been shown to block the increase of fat intake observed after fasting in rat and this behavior is restored after corticosterone treatment (Castonguay, 1991). In addition, chronic GCs treatments in rats have been shown to impair goal-directed behavior as well as motivation to obtain food reward (Gourley et al., 2012).

We previously developed GR^D1Cre^ and GR^DATCre^ mouse models. The GR^D1Cre^ mice are deprived of GR in most of medium spiny neurons and neurons of the basal layers of the cortex (hereafter described as dopaminoceptive neurons) while GR^DATCre^ mice are deprived of GR in dopamine neurons (Ambroggi et al., 2009; Barik et al., 2013). The absence of GR in dopaminoceptive but not dopamine-releasing neurons diminished sensitizing, rewarding, and reinforcing effects of cocaine (Ambroggi et al., 2009; Barik et al., 2010). In striking contrast, we showed that morphine responses in both models remained unaltered (Barik et al., 2010) although stress facilitates opiates effects (Deroche et al., 1995). While these results suggest a GR-dependent dichotomy for the regulation of psychostimulant and opiate responses, such hypothesis still needs to be validated, as GR involvement in responses to other psychostimulant drugs such as amphetamine has never been clearly established. In addition, while extensive work has been made to uncover the role of this receptor in drugs of abuse-related behaviors, its potential contribution in responses to food rewards and the neuronal population that may be involved have yet to be investigated. We therefore, examined the ability of GR along the DA pathway to modulate behavioral responses to amphetamine and food rewards. We demonstrated that GR in dopaminoceptive neurons selectively modulated behavioral and molecular responses to amphetamine without altering rewarding and reinforcing properties of food rewards.

## Materials and methods

### Animal breeding and drug treatments

Nr3c1 (*GR*) gene inactivation was selectively targeted in dopaminoceptive (Nr3c1^loxP/loxP^;(Tg)D1aCre (Lemberger et al., 2007), hereafter designed GR^D1Cre^) or dopamine (Nr3c1^loxP/loxP^;(Tg)BAC-DATiCre*fto* (Turiault et al., 2007), hereafter designed GR^DATCre^) neurons as described in Ambroggi et al. (2009). Experimental animals were obtained by mating Nr3c1^loxP/loxP^ females with either Nr3c1^loxP/loxP^;Tg:D1aCre or Nr3c1^loxP/loxP^;(Tg)BAC-DATiCre*fto* mice. Half of the progeny were mutant animals, the other half were control littermates. Animals were bred and raised under standard animal housing conditions, at 22°C, 55–65% humidity, with a 12-h light/dark cycle (7 am/7 pm) and free access to water and a rodent diet. All experiments were performed in accordance with French (Ministère de l'Agriculture et de la Forêt, 87-848) and the European Directive 2010/63/UE and the recommendation 2007/526/EC for care of laboratory animals. Mice were 2–4 month old males and backcrossed for more than 8 generations on C57BL/6 genetic background. All the experiments have been performed within the hours preceding or encompassing the beginning of dark phase (7 pm), when corticosterone levels are elevated (Le Minh et al., 2001). The behavioral sensitization experiments have been carried out from 6 pm to 9 pm; The CPP experiments from 7 pm to 11 pm and the food progressive ratio (PR) experiment were performed from 4 pm to 6 pm. All drugs were dissolved in saline 0.9%. D-amphetamine (freebase; Sigma-Aldrich, Saint-Quentin Fallavier, France), SKF81297 (salt; Tocris Cookson, Bristol, UK), and MK801 (salt; Tocris Cookson, Bristol, UK) were administered intraperitoneally (ip).

### Locomotor activity and sensitization

Locomotor activity and sensitization were performed as described in Barik et al. (2010). Briefly, locomotor activity was assessed in circular chamber (4.5-cm width, 17-cm external diameter) crossed by four infrared captors (1.5 cm above the base) placed at every 90° (Imetronic, Bordeaux, France). The locomotor activity was counted when animals interrupted two successive beams and thus, had travelled a quarter of the circular corridor. Mice were habituated to the apparatus for 3 h, for 3 consecutive days, and received a saline injection on days 2 and 3. To assess the response to SKF81297 or MK801, on day 4 mice were placed in the apparatus for 90 min before receiving an acute injection of SKF81297 (1.5 or 3 mg/kg) or MK801 (0.2 mg/kg). In the case of amphetamine-induced locomotor sensitization, from day 4 to 8, mice were daily treated with amphetamine or saline after a 90 min habituation to the apparatus. Following 8 days of withdrawal, mice received an acute challenge of amphetamine. The locomotor activity post-injection was acquired for 1 h. For acute drug responses data are recorded as ¼turn per 5 min. For clarity reason, data are presented every 10 min. For locomotor sensitization, data are presented as the sum of activity over 1 h.

### Conditioned place preference

The conditioned place preference (CPP) apparatus consisted of two chambers (20 × 20 × 25 cm) with distinct visual and tactile cues connected by a neutral area. On day 1 (pre-conditioning), mice were placed in the neutral area allowed to freely explore the apparatus for 18 min. The time spent in each chamber was measured. On days 2, 4, 6, and 8, *amphetamine*-paired mice received an *amphetamine* injection (1 or 2 mg/kg) and were confined to one chamber for 25 min. On days 3, 5, 7, and 9, *amphetamine*-paired mice received saline in the opposite chamber and were also confined for 25 min. Saline-paired animals received saline in both chambers. To examine food-induced CPP, mice had limited access to chow pellet in their home-cage for a week, to stabilize their bodyweight to 85% of their original weight. Conditioning for food was similar to that of amphetamine. F*ood-paired mice received* a chow pellet (1 g; standard food CPP) or chocolate with cereals (“chocapic,” Nestlé, 0.5 g; palatable food CPP) in the paired chamber on days 2, 4, 6, 8 and confined for 30 min. On days 3, 5, 7, 9, mice were confined to the other, unpaired, chamber but had no access to food. No food-paired mice were alternatively placed in each chamber with no access to food at any time. During the post-conditioning (day 10), mice, in absence of any reward, were allowed to freely explore both chambers for 18 min. The CPP scores were expressed as the increase of time spent in the paired chamber between the post- and the pre-conditioning sessions.

### Progressive ratio for food

#### Apparatus

The PR experiment took place in 12 home cages containing an operant conditioning wall (24 × 28 × 28 cm, Operant Behavior System, TSE, Bad Homburg, Germany). The operant wall had two retractable levers, a food pellet dispenser delivering 20 mg sucrose pellets with peanut butter flavor (GlaxoSmithKline, TestDiet, Richmond, IN, USA) and white light bulbs above the levers and in the dispenser. The operant walls were covered outside periods of training and testing. The boxes were covered with a layer of corn cob bedding and enriched with cotton nest pads. Water was available *ad-libitum*. During the period of habituation food (chow, SAFE, Augy, France) was also available *ad-libitum*.

#### Habituation, training, and testing

Mice were maintained at 85% of their initial body weight during training and testing. We tested 6 control and 6 GR^D1Cre^ mice. During the habituation period (2 days) mice were placed in the operant boxes with *ad-libitum* access to food. The mice had continuously access to the operant wall and learnt to lever press for sucrose pellets under a fixed ratio 1 (FR1) schedule (i.e., a single press on the active lever resulted in the delivery of one sucrose pellet). Mice were trained on a FR1 schedule overnight. for 4 days. The FR1 schedule was followed by 6 days of PR schedule during which the cost of a reward is progressively increased for each following reward in order to determine the amount of work the mouse is willing to put into obtaining the reward. The response requirement increases incrementally according to a non-arithmetic progression: 1, 2, 2, 3, 3, 3, 4, 4, 4, 4, 5, … etc. and forms the following series: 1, 2, 4, 6, 9, 12, 15, 19, 23, 27, 31, … etc. PR sessions were carried out once a day. One non-responding control mouse was excluded from the analysis. Breaking point values were defined as the last ratio completed by the animal followed by 15 min during which no additional reward was earned.

### Immunohistochemistry

Immunohistochemistry was performed as described in Barik et al. (2010). Briefly, mice were deeply anaesthetized with pentobarbital (Centravet, France) and transcardially perfused with cold phosphate buffer (PB: 0.1 M Na_2_HPO_4_/NaH_2_PO_4_, pH 7.4), followed by 4% PFA in PB. Brains were post-fixed overnight in 4% PFA-PB. Free-floating vibratome sections (30 μm) were rinsed twice with PBS (20 min) and incubated (30 min) in PBS-BT (PBS 0.5% BSA, 0.1% Triton X-100) with 10% normal goat serum (NGS). Sections were incubated (4°C) in PBS-BT, 1% NGS, with rabbit anti-c-Fos (1:500, Abcam, Cambridge, MA) for 36 h. Sections were rinsed in PBS and incubated (2 h) in goat anti-rabbit biotinylated secondary antibody (1:1000, Vector Laboratories, Burlingame, CA) in PBS-BT, 1% NGS. PBS-rinsed sections were incubated in avidin-biotin-peroxydase complex (ABC reagent; Vector Laboratories, 1:1000) for 1 h. Signal was revealed using the peroxidase-substrate-kit-DAB, as recommended by Vector Laboratories. Quantification of c-Fos immunopositive cells was done semi-automatically using Mercator Explora-Nova software (La-Rochelle, France). CPU and NAc regions were delineated according to Paxino's mouse brain atlas. For, drug-induced c-Fos expression, mice received an acute challenge of saline or amphetamine, and were perfused 1 h later.

### Micro-iontophoresis and *in vivo* recordings

Electrophysiological recordings of NAc medium spiny neurons were performed during the diurnal phase. The experimenter was blind to the genotype during recordings. Mice were anesthetized with chloral hydrate (5.0 mg/kg, i.p.) and mounted in a stereotaxic apparatus. The lateral tail vein was catheterized to administer additional anesthetic or drugs. Body temperature was monitored and maintained with a heating pad at 36.5–37.0°C. Standard electrophysiological procedures were employed. The electrode signal was amplified 2000 times with an AC high impedance amplifier, band pass filtered at 0.4–1 kHz (Dagan 2400A, Minneapolis, MN), and digitized with an interface board at 10 kHz (Digidata 1440A, Axon Instruments Inc., Foster City, CA) and fed to a computer for offline analysis.

For single unit recordings of NAc medium spiny neurons, five barrels manufactured electrodes (ASI instruments, Warren, MI) were pulled and broken to a tip diameter of 8–15 μm. The center barrel was filled with 2 M NaCl containing 1% Fast Green dye (impedance 2–6 MΩ) and was used to record neuronal activity. One side barrel (impedance 20–60 MΩ) was filled with 150 mM NaCl for automatic current balancing. The other barrels were filled with L-glutamate (100 mM, pH 8), which was ejected as an anion. A retaining current (5–10 nA) was applied during non-ejection periods to minimize passive diffusion.

Electrodes were lowered in the NAc as followed: AP+1.1/+1.7, L+0.6/1.2 and DV−3.9/−5.0 mm from the cortical surface. Because most NAc neurons are quiescent in the basal state, glutamate was ejected by micro-iontophoresis while searching for neurons. Once a neuron was detected, the stability of the signal to noise ratio and waveform characteristics were assessed. Recorded neurons were identified as medium spiny neurons NAc neurons by their anatomical location and waveform durations comprised between 1.1 and 1.8 ms (White, 1996; Kish et al., 1999; Mallet et al., 2005). To generate current-response curves, glutamate was ejected by micro-iontophoresis using escalating currents applied in 15 s pulses interspersed with 15 s of non-ejection periods.

### Statistics

Data are presented as means ± s.e.m. Statistical analysis was carried out using Two-Way analysis of variance (ANOVA) for CPP, drug-elicited c-Fos induction. Acute locomotor responses and locomotor sensitization experiments were analyzed with Three-Way ANOVA with repeated measures. *Post-hoc* Bonferroni's test or Dunnett's for multiple comparison tests were used when appropriate.

## Results

### Acute neuronal and behavioral responses to amphetamine in mice deprived of GR gene within the meso-cortico-limbic dopamine system

**Figure 1:**
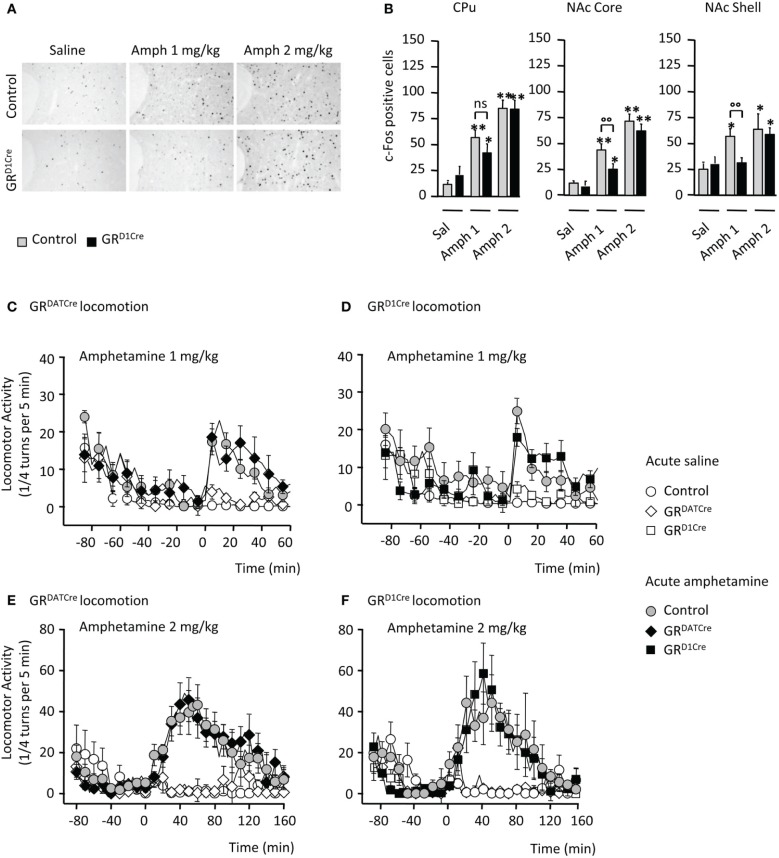
**Impaired molecular but not locomotor responses following an acute amphetamine challenge in GR^D1Cre^ mice**. **(A)** Representative example of c-Fos induction in the NAc core of a control and a GR^D1Cre^ mouse in response to saline, amphetamine 1 and 2 mg/kg. **(B)** Amphetamine-induced c-Fos expression in the caudate-putamen (left panel), the nucleus accumbens core (middle panel), and shell (right panel) of control and GR^D1Cre^ mice. *n* = 4–8 animals per group; saline vs. drug: ^*^P < 0.05; ^**^P < 0.01; control vs. mutant: °°*P* < 0.01. Locomotor activity is expressed as the sum of ¼ turns in a circular cylinder per 5 min following acute drug (gray or black) or saline (white) injections in control (circles), GR^DATCre^ (diamonds), and GR^1Cre^ (squares) mice. **(C)** Similar locomotor response to a single injection of saline and amphetamine (1 mg/kg) in control and GR^DATCre^ mice. Interaction Drug × Time *F*_(29, 840)_ = 7.9, *P* < 0.001, with no genotype effect *F*_(1, 420)_ = 1.3, *P* > 0.05. **(D)** Control and GR^D1Cre^ mice equally respond to an acute 1 mg/kg of amphetamine. Interaction Drug × Time *F*_(87, 780)_ = 1.7, *P* < 0.001, with no genotype effect *F*_(1, 656)_ = 0.1, *P* > 0.05. **(E,F)** Amphetamine (2 mg/kg) induced a robust increase in locomotor response regardless of the genotype in control and GR^DATCre^ mice [**(E)**, no genotype effect *F*_(1, 637)_ = 0.3, *P* > 0.05] and control and GR^D1Cre^ mice [**(F)**, no genotype effect *F*_(1, 686)_ = 0.8, *P* > 0.05].

We studied neuronal activation upon amphetamine response by quantifying c-Fos expression in mutant and control littermates. Consistent with previous findings (Moratalla et al., 1996), amphetamine (1 and 2 mg/kg) elicited a significant increase in the number of c-Fos-positive cells within the CPu and the NAc core and shell of control animals (Figures 1A,B). This effect was significantly diminished within the NAc subdivisions and displayed a trend toward a decrease in the CPu when GR^D1Cre^ mice were administered 1 mg/kg of the drug (Figures 1A,B). No significant genotype difference was observed when animals were administrated a higher dose (2 mg/kg) of amphetamine (Figures 1A,B). These results indicate a hyporesponsiveness of the NAc of GR^D1Cre^ mice to low doses of amphetamine.

In many species including rodents, psychostimulant injection triggers a typical increase in locomotor responses. Thus, locomotor activity of GR^D1Cre^ mice and their control littermates was measured following acute amphetamine administration. To ascertain the lack of involvement of GR in dopamine-releasing neurons we also examined responses in GR^DATCre^ mice and their respective controls. While saline injection failed to produce any locomotor hyperactivity, amphetamine increased locomotor activity in control mice with a stronger response at 2 mg/kg compared to 1 mg/kg (Figures 1C–F). The locomotor response to single amphetamine injection was the same in both GR^DATCre^ (Figures 1C,D) and GR^D1Cre^ mice (Figures 1E,F) compared to their respective control littermates, for both doses tested. Thus, the absence of GR in dopaminoceptive neurons does not alter the acute behavioral response to amphetamine.

### The absence of GR in dopaminoceptive neurons decreases the sensitivity to locomotor sensitizing properties of amphetamine

**Figure 2:**
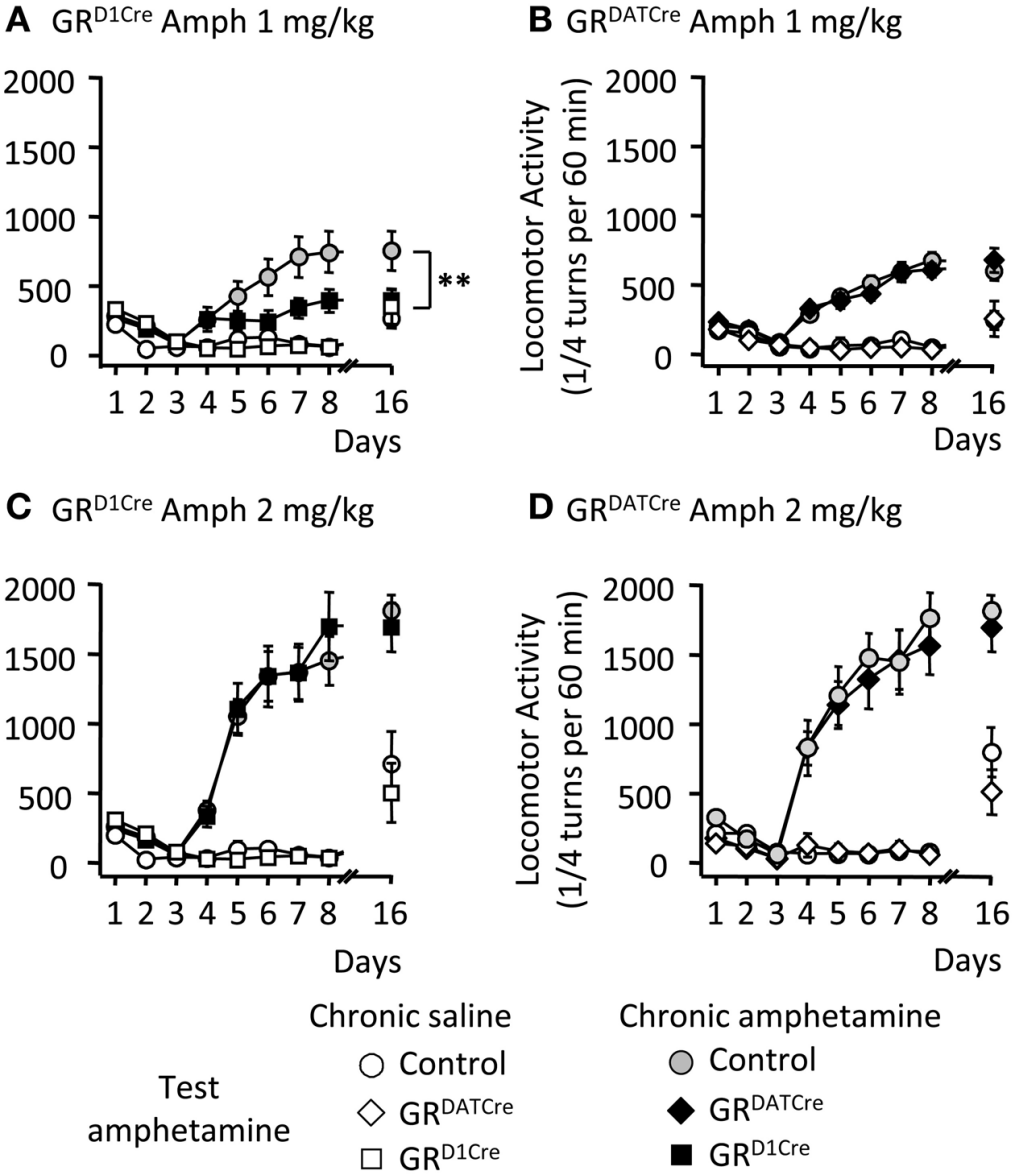
**The locomotor sensitization to low dose of amphetamine is selectively abolished in GR^D1Cre^ mice**. Locomotor activity is expressed as the sum of ¼ turns in a circular cylinder per hour following repeated drug or saline injections. **(A)** Locomotor sensitization to 1 mg/kg amphetamine daily injections in control (gray circles) and GR^D1Cre^ mice (black squares). White circles and white squares represent control and GR^D1Cre^ mice, respectively, which received daily injections of saline followed by a challenge injection of amphetamine 1 mg/kg on the test day. Amphetamine (1 mg/kg) induced locomotor sensitization that was abolished in GR^D1Cre^ mice at day 16; interaction Genotype × Treatment *F*_(1, 28)_ = 5.2; saline vs. drug: *P* < 0.01; control vs. mutant: ^**^P < 0.01. **(B)** Locomotor sensitization to 1 mg/kg amphetamine daily injections in control (gray circles) and GR^DATCre^ (black diamond) mice. White circles and white squares represent control and GR^DATCre^ mice, respectively, which received daily injections of saline followed by a challenge injection of amphetamine 1 mg/kg following an 8-day withdrawal period. Interaction Genotype × Treatment *F*_(1, 33)_ = 0.16; saline vs. drug: *P* < 0.001; control vs. mutant: *P* = 0.6. **(C,D)** are same than **(A,B)**, respectively, but with 2 mg/kg amphetamine. *n* = 8–10 per group. **(C)** Interaction Genotype × Treatment *F*_(1, 36)_ = 1.72; saline vs. drug: *P* < 0.001; control vs. mutant: *P* = 0.67. **(D)** Interaction Genotype × Treatment *F*_(1, 37)_ = 0.27; saline vs. drug: *P* < 0.001; control vs. mutant: *P* = 0.21.

One of the key features of abused drugs is their ability to trigger locomotor sensitization (Vanderschuren and Pierce, 2010), i.e., a progressive and enduring augmentation in locomotor activity following repeated drug injection. We assessed the sensitizing properties of amphetamine in GR^D1Cre^ and GR^DATCre^ mice, and respective control littermates. Five consecutive daily injections of amphetamine (1 mg/kg), but not saline, induced significant locomotor sensitization in control mice, which was still persistent following an 8-day withdrawal period (Figures 2A,B). In contrast GR^D1Cre^ mice failed to develop locomotor sensitization (Figure 2A) whereas the absence of GR in dopamine neurons (GR^DATCre^ mice) had no effect (Figure 2B). When tested at a higher dose (2 mg/kg), amphetamine induced a more robust locomotor sensitization that was similar in both mutant lines and their respective control littermates (Figures 2C,D). Hence, GR within dopaminoceptive neurons is selectively required for enabling locomotor sensitization to low doses of amphetamine. This suggests that elevated doses of amphetamine are likely to provoke stronger molecular activations that hence may overcome GR's modulatory effects.

### The absence of GR in dopaminoceptive neurons decreases the sensitivity to rewarding properties of amphetamine

**Figure 3:**
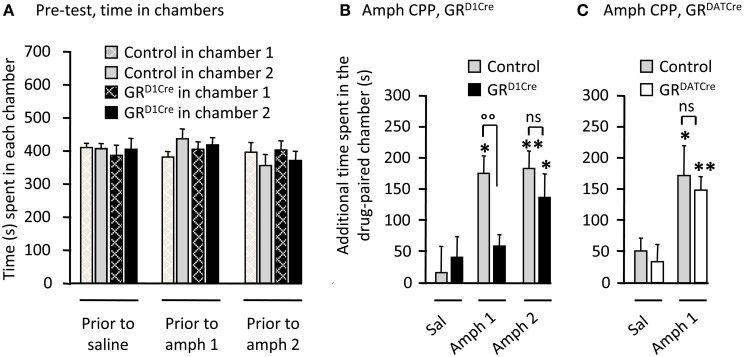
**GR^D1Cre^ mice show a decreased sensitivity to amphetamine rewarding properties**. CPP scores represent the time difference between post-conditioning and pre-conditioning phases that mice spent in the reward-paired chamber. **(A)** Time spent in each chamber of the CPP apparatus by control (gray bars) and GR^D1Cre^ mice (black bars), during the pre-conditioning phase. **(B)** CPP to amphetamine 1 and 2 mg/kg in control and GR^D1Cre^ mice. CPP induced by amphetamine (1 mg/kg) differed in control and GR^D1Cre^ mice. Interaction Drug × Genotype *F*_(1, 39)_ = 4.5, *P* < 0.05. **(C)** Amphetamine (1 mg/kg)-induced comparable CPP in both control and GR^DATCre^ mice. No interaction Drug × Genotype *F*_(1, 35)_ = 1.1, *P* > 0.05. ns: non-significant, ^*^P < 0.05; ^**^P < 0.01; control vs. mutant: °°*P* < 0.01. *n* = 8–12 mice per group.

Repeated pairings of abused drugs in a specific environment triggers reward-associated memories (Kelley, 2004) thought to reflect changes in the motivational state of the subject (Bardo and Bevins, 2000). We next studied amphetamine CPP, a commonly employed context-dependent paradigm, to measure the effects of rewarding stimuli in GR mutant animals. On the pre-conditioning day, all four groups of animals spent similar amount of time in the two distinct chambers (Figure 3A). Pairing injections of 1 or 2 mg/kg of amphetamine produced a significant CPP in control mice (Figures 3B,C). Mirroring the results obtained for locomotor sensitization, these rewarding effects were abolished in GR^D1Cre^ mice at the lowest dose of amphetamine tested, but were not significantly different from controls when the dose was increased up to 2 mg/kg (Figure 3B). The absence of GR in pre-synaptic dopamine neurons had no effect as GR^DATCre^ mice displayed normal CPP to 1 mg/kg amphetamine (Figure 3C).

### Abnormal locomotor response to NMDA antagonist in absence of GR in dopaminoceptive neurons

**Figure 4:**
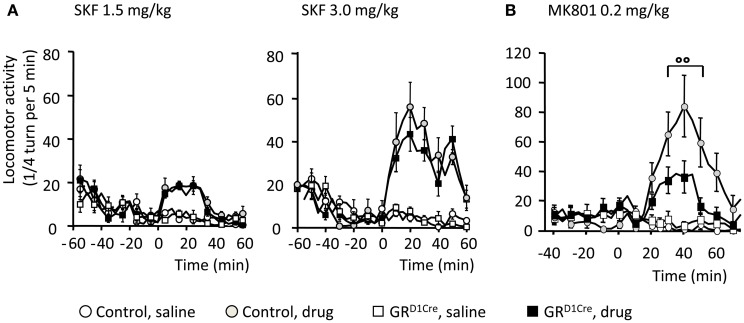
**Normal D1-like dopamine receptor agonist induced locomotor activity, but impaired MK801-elicited hyperlocomotion in GR^D1Cre^ mice**. Locomotor responses are presented as ¼ turn per 5 min. **(A)** Locomotor response to saline and SKF81297 1.5 mg/kg (left panel) and 3 mg/kg (right panel) in control and GR^D1Cre^ mice. Interaction Drug × Time for SKF81297 at 1.5 mg/kg [*F*_(17, 714)_ = 10.3, *P* < 0.001] and 3 mg/kg [*F*_(17, 714)_ = 9.4, *P* < 0.001], but no interaction Drug × Time × Genotype, *F*_(17, 714)_ = 1.2, *P* > 0.05 and *F*_(17, 714)_ = 0.8, *P* > 0.05, respectively. **(B)** MK801 elicited a stronger hyperlocomotion in control than GR^D1Cre^ mice, interaction Drug × Time × Genotype, *F*_(18, 756)_ = 1.9, *P* < 0.01. °°*P* < 0.01, control vs. mutant. *n* = 8–14 mice per group.

In response to abused drugs, the increase of dopamine release within the CPu and NAc is thought to filter and selectively reinforce connections arising from excitatory corticostriatal projections (Bamford et al., 2004). Hence this dopamine/glutamate interaction is key to shape medium spiny neurons responsiveness at both electrophysiological and molecular levels, with a central implication of D1 dopamine receptors and NMDA glutamate receptors in these processes (Nicola et al., 2000; Pascoli et al., 2011). We therefore, examined whether GR gene inactivation within dopaminoceptive neurons could impact on dopamine and glutamate receptor functions that may explain the observed phenotype. To challenge D1 dopamine receptor, we injected SKF81297, a selective D1-like receptor agonist and measured subsequent locomotor responses. As previously reported (Corvol et al., 2007), acute systemic SKF81297 injection elicited hyperlocomotion in control animals (Figure 4A). At the 2 doses examined (1.5 and 3 mg/kg), GR^D1Cre^ mice did not differ from their respective control littermates (Figure 4A) ruling out an impaired functionality of D1 dopamine receptors. To determine the state of glutamate transmission in GR^D1Cre^ mice, we then assessed the ability of MK801, a non-competitive NMDA antagonist, to elicit hyperlocomotion (Qi et al., 2008). Systemic injection of MK801 (0.2 mg/kg) triggered a robust hyperlocomotion in controls that was significantly decreased in mutant mice (Figure 4B). Therefore, this set of experiments suggests that the impaired glutamate response may play a role in the diminished response to amphetamine.

### Decreased responsiveness to glutamate in a subpopulation of medium spiny neurons within the nucleus accumbens of GR^D1Cre^ mice

**Figure 5:**
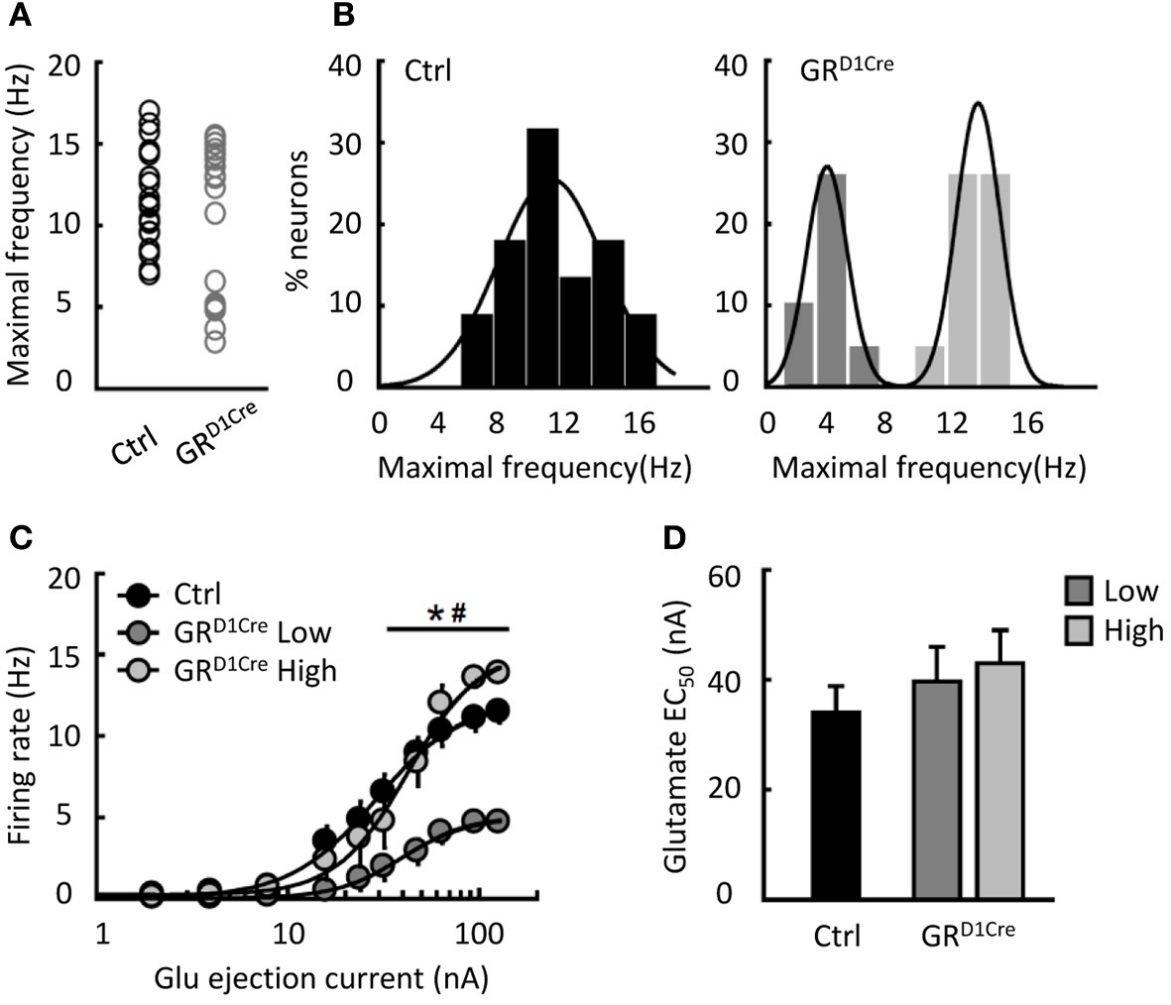
**Reduced glutamate induced-firing in a sub-population of NAc neurons in GR^D1aCre^ mice**. **(A)** Individual maximal frequencies reached after micro-iontophoretic application of glutamate in NAc neurons (*n* = 22 cells in 13 control mice and *n* = 20 in 11 GR^D1Cre^ mice). **(B)** The distribution of maximal frequencies after glutamate application was normal in control mice (Shapiro–Wilk test, *W* = 0.96, *p* = 0.58) and bimodally distributed in GR^D1Cre^ mice (Shapiro–Wilk test, *W* = 0.82, *p* < 0.002). **(C)** Glutamate dose response in NAc neurons from control mice (black bars) and in the two populations of NAc neurons found in GR^D1Cre^ mice. Slow-firing neurons recorded in GR^D1Cre^ mice differed from the two other populations [dose population interaction *F*_(20, 390)_ = 5.47, *p* < 0.0001]. ^*^P < 0.001 for GR^D1Cre^ slow vs. GR^D1Cre^ high, ^#^*P* < 0.001 for GR^D1Cre^ slow vs. control. **(D)** The EC_50_ of the 3 populations of neurons did not differ [*F*_(2, 39)_ = 0.72, *p* = 0.49].

Altered glutamatergic neurotransmission within the NAc might contribute to the impaired behavioral responses to amphetamine and MK801 as well as to the decrease of accumbal c-Fos induction observed in GR^D1Cre^ mice. To investigate the functional effects of GR inactivation on glutamatergic neurotransmission within the NAc, we analyzed the reactivity to glutamate of NAc medium spiny neurons. We performed *in-vivo* recordings of NAc medium spiny neurons coupled to glutamate micro-iontophoresis in control and GR^D1Cre^ mice. For each neuron, incremental glutamate ejection currents were applied until the neuron reached its maximal firing frequency. In control mice, the maximal frequencies observed were normally-distributed, ranging from 7 to 17 Hz (Figures 5A–C). In contrast in GR^D1Cre^ mice, the distribution was bimodal: one population had maximal frequencies in a range similar to that observed in control mice, whereas another population was shifted toward lower frequencies; these neurons were unable to fire above 6 Hz. The analysis of the dose-response functions revealed that the EC_50_ was similar between controls, fast and slow neurons (Figure 5D). We found no evidence of anatomical segregation of fast and slow neurons; in particular, they were found equally in the core and the shell (*P* > 0.05). This experiment shows that GR positively controls the reactivity to glutamate of a subset of NAc medium spiny neurons.

### Unaltered responses to food rewards in GR^D1Cre^ mice

**Figure 6:**
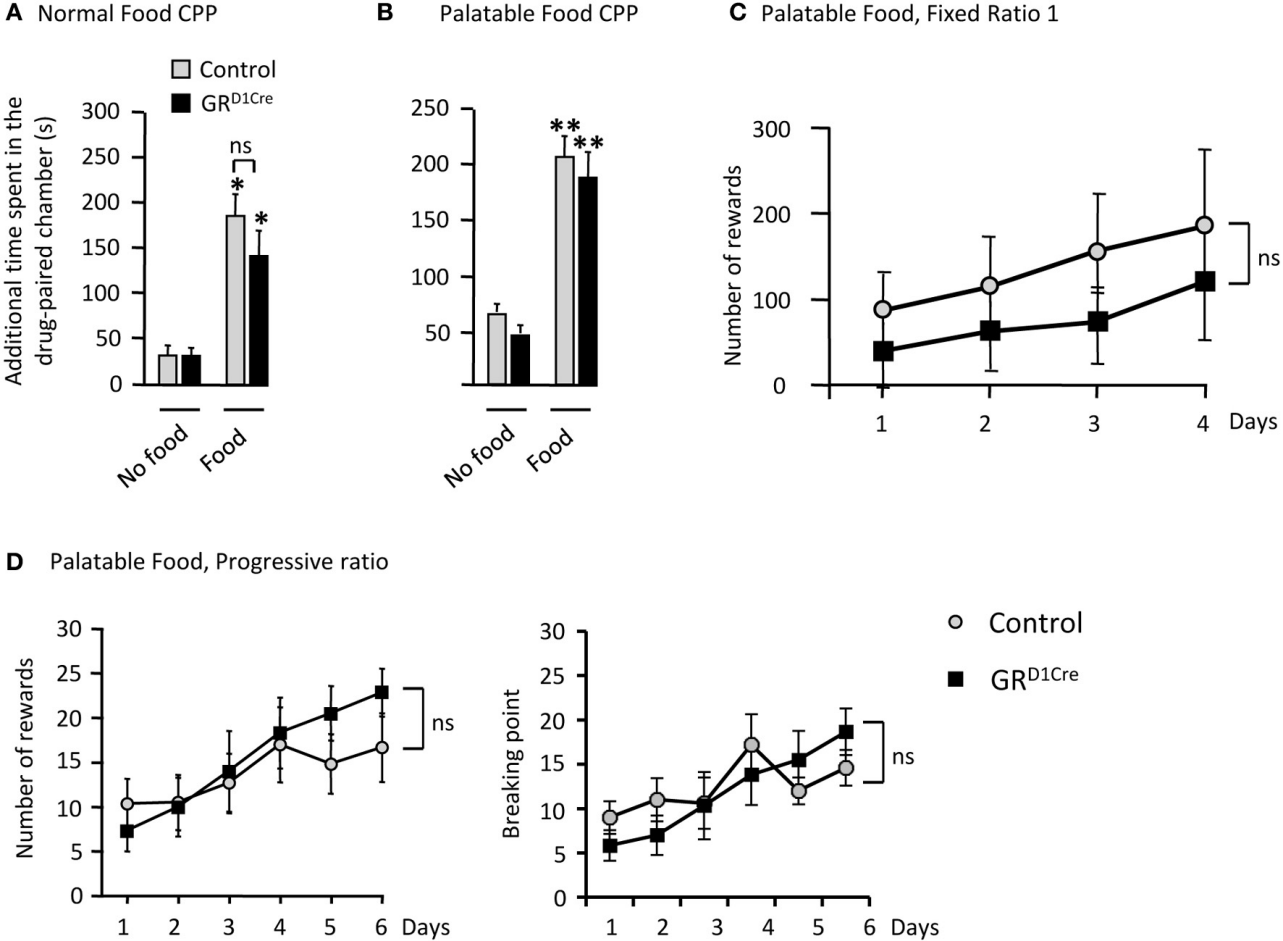
**Unaltered responses to food reward in GR^D1Cre^ mice**. CPP scores represent the time difference between post-conditioning and pre-conditioning phases that mice spent in the reward-paired chamber. **(A)** Control and GR^D1Cre^ mice show similar levels of CPP when normal chow pellet were use as a conditioning stimulus. Effect of Food *F*_(1, 33)_ = 4.4, *P* < 0.05, but no effect of genotype *F*_(1, 33)_ = 0.9, *P* > 0.05. **(B)** Similar responses were also obtained when control and GR^D1Cre^ mice were paired with palatable food, which significantly increased the time spent in the paired chamber [*F*_(1, 35)_ = 45.8, *P* < 0.001] regardless of the genotype [*F*_(1, 35)_ = 0.9, *P* > 0.05]. **(C)** In operant chambers, both control and GR^D1Cre^ mice exhibited similar responses for palatable food reward either under a fixed ratio 1 schedule [no effect of genotype *F*_(1, 40)_ = 0.8, *P* > 0.05], or **(D)** a more stringent progressive ratio schedule [no effect of genotype, *F*_(1, 60)_ = 0.9, *P* > 0.05]. For CPP *n* = 8–12 mice per group and for operant responding for palatable food *n* = 6 mice per group. ns: non-significant ^*^P < 0.05, ^**^P < 0.01.

As the processing of natural rewards and addictive drugs activate overlapping pathways, we sought to determine whether GR gene inactivation in dopaminoceptive neurons resulted in a general impairment of natural reward-seeking. As we did for amphetamine, we first tested control and mutant mice in two CPP experiments in response to normal (chow pellets) or palatable (chocolate) food. Mice were exposed for 30 min to either food in the paired chamber and spent the same amount of time, without food, in the opposite (unpaired) chamber on alternate days. Following 8 days of conditioning, the time increase in the paired chamber was used as an index of place preference. Both normal and palatable food elicited significant CPP in control animals. However, unlike our results with amphetamine, CPP remained unaltered in GR^D1Cre^ mice (Figures 6A,B respectively).

Next, as GR inactivation within dopaminoceptive neurons has been reported to decrease motivation for cocaine in a PR schedule (Ambroggi et al., 2009), we thus, tested motivation of GR^D1Cre^ mice to respond instrumentally for food rewards. During initial instrumental training, under a fixed-ratio1 schedule, control and GR^D1Cre^ mice did not show significant differences in the number of responses (Figure 6C) and ate similar amount of pellets (control: 6.04 ± 0.29 g, GR^D1Cre^: 6.3 ± 0.23 g). During the learning phase of the PR schedule (where the response-requirement increased after each reward obtained), control and mutant mice exhibited a comparable increase in their responding for food (Figure 6D left panel). Analysis of the breaking points (defined as the last ratio completed by the animal followed by 15 min during which no additional reward was earned) revealed no difference between controls and GR^D1Cre^ mice (Figure 6D right panel). While during initial instrumental training GR^D1Cre^ mice showed a trend toward a decrease in instrumental responses compared to controls, the opposite was rather observed during the last two sessions of PR. This set of data suggests that GR in dopaminoceptive neurons does not modulate food reward responses.

## Discussion

In this study, we aimed at dissecting the modulatory role of GR within the meso-cortico-limbic dopamine system, on responses to amphetamine and food rewards. We showed that inactivation of GR gene in dopaminoceptive cells, but not in dopamine cells, decrease amphetamine-mediated locomotor sensitization and CPP, two behavioral features of psychostimulants. Along with these behavioral deficits, absence of GR in dopaminoceptive cells decreased the post-synaptic response to amphetamine within the NAc as assessed by c-Fos immunostaining. These changes in behavioral and post-synaptic neuronal activation to amphetamine may involve abnormal glutamate transmission as mice deprived of GR in dopaminoceptive neurons showed a decrease in locomotor response to NMDA receptor antagonist MK801 and a decrease in neuronal response to intra-accumbal glutamate administration. These results extend our previous findings which showed that inactivation of GR in the same cell population dampens behavioral and molecular responses to cocaine, another psychostimulant drug (Ambroggi et al., 2009; Barik et al., 2010). These modulatory effects of GR appear to be selective to psychostimulants as neither morphine (Barik et al., 2010) nor food reward responses (the present study) are affected by the inactivation of *GR* gene in the meso-cortico-limbic dopamine system.

While a body of evidence suggest that stress reaction, as well as GCs, facilitate behavioral responses to amphetamine, the brain regions targeted by GCs actions remained to be identified. Furthermore, the determination of the receptor type involved is still a matter of debate. Although pharmacological antagonism of GR, using the antagonist RU486, has been shown to decrease amphetamine-induced locomotor sensitization without changing the acute locomotor response to the drug (De Vries et al., 1996), systemic administration of GR agonist dexamethasone decreased amphetamine induced hyperactivity (Capasso et al., 1996). These confounding results are however difficult to interpret as the RU486 is also a potent progesterone receptor antagonist (Cadepond et al., 1997), and dexamethasone, when injected systemically, is actively expelled from the brain compartment, hence substantially limiting its effects (Meijer et al., 1998). Systemic dexamethasone may have resulted in a depletion of endogenous GCs levels via the negative feedback exerted by activation of GR in the pituitary gland. The response to amphetamine has also been studied in a transgenic mouse model expressing a neurofilament promoter-driven antisense RNA complementary to a fragment of cDNA that codes for the mouse GR. In this model, GR mRNA levels are decreased by 50% on average in the brain (Pepin et al., 1992). An enhanced locomotor response to amphetamine in this model suggested that GR may decrease sensitivity to this drug (Cyr et al., 2001). However, disturbances in HPA axis function, including elevated levels of adrenocorticotropin hormone and GCs and a lack of diurnal variation in HPA axis activity may have led to confounding effects (Beaulieu et al., 1994). The fact that selective ablation of GR from dopamine neurons had no effect on behavioral responses to amphetamine is in coherence with the absence of effects we previously observed on behavioral responses to cocaine and on spontaneous firing of dopamine neurons (Ambroggi et al., 2009; Barik et al., 2010). We believe that the reduced behavioral responses to amphetamine observed in GR^D1Cre^ mice result from the absence of GR in dopaminoceptive neurons. Indeed, although expression of D1 receptor have been reported in peripheral tissues, (Ozono et al., 1997) the potential GR gene recombination in the periphery in GR^D1Cre^ mice does not alter HPA-axis activity (Ambroggi et al., 2009) and is unlikely to alter amphetamine metabolism.

Strikingly, GR in dopaminoceptive neurons appears to modulate behavioral responses to low (1 mg/kg) but not high (2 mg/kg) doses of amphetamine. This effect has been observed for both locomotor sensitization and CPP. In rats, it was reported that the environmental changes in housing conditions, which differently shape the HPA axis, only affected the reinforcing properties of low doses of amphetamine, suggesting that the environment modifies the threshold for positive hedonic effects of amphetamine (Bardo et al., 2001; Green et al., 2002; Stairs et al., 2011). Altogether, these data suggest that stress-induced GCs release increases the sensitivity to reinforcing, rewarding, and sensitizing properties of amphetamine for moderate doses and this effect could be at least partially mediated through activation of GR within dopamine-targeted areas. In addition to these behavioral effects, we also observed a decrease in the induction of c-Fos by amphetamine specifically within the NAc of GR^D1Cre^ mice. An acute cocaine injection has been previously shown to predominantly (but not exclusively) induce c-Fos in D1-expressing medium spiny neurons (Bertran-Gonzalez et al., 2008). We might expect a similar pattern of induction in response to amphetamine. Despite the decreased neuronal response to amphetamine, their acute locomotor response was unaltered compared to controls. Such apparent contradiction can however be partially resolved. A previous study showed that complete absence of c-Fos in D1-expressing neurons, obtained by conditional gene inactivation, does not alter the marked locomotor response to an acute injection of the D1-like agonist SKF81297. It also has no effect on the acute locomotor response to a moderate (10 mg/kg) dose of cocaine but does impair locomotor sensitization (Zhang et al., 2006). Thus, c-Fos induction in dopamine-innervated areas is not necessary to build an acute locomotor response to moderate doses of psychostimulant drugs but seems crucial for the development of sensitizing effects. In the present study we did not investigate for c-Fos induction after repeated injection of amphetamine. However, we have previously shown that repeated administration of cocaine leads to a sensitization of c-Fos mRNA induction specifically in the CPu and motor cortex and that this sensitization was abolished in mice lacking GR in the whole central nervous system (Deroche-Gamonet et al., 2003).

Decreased behavioral and post-synaptic responses to amphetamine observed in GR^D1Cre^ mice were unlikely due to alterations in post-synaptic dopamine D1-mediated signaling as these mice showed similar responses to dopamine D1-like receptor agonists SKF81297 compared to controls. In contrast, we showed that the absence of GR in dopaminoceptive neurons dampened locomotor responses to MK801, a NMDA receptor antagonist. Given that c-Fos induction by amphetamine is specifically dampened in the NAc, this suggests that glutamate neurotransmission could potentially be impaired within this brain region. This is confirmed by our electrophysiological data. In GR^D1Cre^ mice, about half of NAc medium spiny neurons were found to be less reactive to glutamate while the remaining neurons had normal responses. Medium spiny neurons in both CPu and NAc can be segregated in two neuronal populations expressing either D1 or D2 dopamine receptors with some overlap between these two populations (Bertran-Gonzalez et al., 2010). The bimodal distribution could therefore be the result of this segregation. However, GR^D1Cre^ mice are likely to be recombined in both populations (Barik et al., 2013), potentially because of transient developmental expression of the D1 receptor. GR may differentially regulate glutamate reactivity in D1- or D2-expressing neurons and future studies will be needed to directly test this hypothesis. The alteration of glutamate neurotransmission within the NAc could explain the decreased responses to psychostimulants, which also largely relies on glutamate transmission (Kalivas, 2000). A body of evidence suggests that GR can modulate glutamate transmission in the brain (Popoli et al., 2012). In addition, in a previous study, microarray and RT-qPCR revealed changes in the expression of NMDA receptor subunits and in AMPA/kainate signaling pathways within the striatum of GR^D1Cre^ mice (Barik et al., 2010). These changes could account for the alteration of glutamate neurotransmission in these mice.

This study, as well as our previous work, clearly demonstrate that the absence of GR in dopaminoceptive neurons diminish reinforcing, rewarding, and sensitizing effects of psychostimulants. As the same brain circuits are involved in behavioral responses to abused drugs and natural rewards, we also investigated the effect of inactivating GR within dopaminoceptive cells on the hedonic reactions to food reward (either regular chow or palatable food) in a CPP paradigm and on the willingness to expend effort to obtain a food reward in a progressive ratio task. Both meso-accumbens and nigro-striatal dopamine pathways have been involved in the modulation of motivation and decision-making processes essential to reach a goal (Schultz, 2006; Wise, 2008). Moreover, deficits in motivation are hallmark features of many psychiatric disorders including depression for which stress exposure is an important environmental risk factor. A recent study in rat showed that chronic stress exposure impairs the sensitivity to changes in outcome value and in response-outcome contingency suggesting that chronic stress exposure might induce deficits in reward expectation (Dias-Ferreira et al., 2009). Along with these deficits, structural changes in prefrontal areas and in the dorsal striatum have been observed suggesting that stress exposure may lead to goal-directed behavior impairments by altering cortico-striatal circuits (Dias-Ferreira et al., 2009). Interestingly, these results were mimicked by chronic GCs administration. Indeed, chronic GCs treatments have been shown to impair goal-directed response-outcome associations as well as motivation to obtain food reward in a PR schedule. On the other side, acute pharmacological blockade of GR only impaired response-outcome association sparing motivation (Gourley et al., 2012). Surprisingly, we did not find any difference between GR^D1Cre^ mice and control littermates in both CPP and PR tasks suggesting that GR in this cell population may not be necessary to modulate rewarding and reinforcing properties of food as it is for psychostimulant drugs. In GR^D1Cre^ mice, most of striatal and NAc neurons show an inactivation of GR. Indeed, GR ablation was observed in more than 85% of striatal neurons. However, within the cortex, only neurons located within deep layers (V/VI) exhibit a percentage of recombination comparable to that of striatal neurons, while most of neurons from upper layers still express GR (Barik et al., 2013). Thus, the effects of stress and GCs on goal-directed behavior and motivation might be rather mediated by an impact at the level of the PFC rather than the striatum or the NAc. Another possibility is that the inactivation of GR in dopaminoceptive neurons may protect from deleterious effects of chronic stress exposure rather than having an effect at basal stress levels. Further studies will be required to explore these hypotheses.

Our findings along with previous studies show that GR in dopaminoceptive neurons selectively modulates reinforcing, rewarding, and sensitizing properties of psychostimulant drugs such as cocaine and amphetamine. These effects seem mediated by alterations of integration of glutamate signaling within the striatum and NAc. On the other side, behavioral responses to food remained unchanged in the absence of GR within dopaminoceptive neurons. These results could be interesting in the context of the development of new medications able to decrease sensitivity to abused drugs while sparing general motivation for natural reinforcers.

## Author contributions

Sébastien Parnaudeau, François Tronche, and Jacques Barik designed the study. Sébastien Parnaudeau, Frédéric Ambroggi, Marc Turiault, Marie-louise Dongelmans, Anne-Sophie Delbes, Céline Cansell, Serge Luquet, and Jacques Barik performed research. Sébastien Parnaudeau, Frédéric Ambroggi, Marc Turiault, Marie-louise Dongelmans, Pier-Vincenzo Piazza, Jacques Barik, and François Tronche analyzed the data. Sébastien Parnaudeau, Frédéric Ambroggi, François Tronche, and Jacques Barik wrote the manuscript.

### Conflict of interest statement

The authors declare that the research was conducted in the absence of any commercial or financial relationships that could be construed as a potential conflict of interest.
